# Characterization of *FOLH1* Expression in Renal Cell Carcinoma

**DOI:** 10.3390/cancers16101855

**Published:** 2024-05-13

**Authors:** Eric Ovruchesky, Elizabeth Pan, Melis Guer, Andrew Elliott, Shankar Siva, Praful Ravi, Bradley McGregor, Aditya Bagrodia, Ithaar Derweesh, Pedro Barata, Elisabeth I. Heath, Emmanuel S. Antonarakis, Sourat Darabi, Dave S. B. Hoon, Amir Mortazavi, Toni K. Choueiri, Chadi Nabhan, Shuanzeng Wei, Rana R. McKay

**Affiliations:** 1 Division of Urologic Oncology, Department of Oncology, Moores Cancer Center, University of California San Diego, San Diego, CA 92037, USA; 2 Department of Clinical and Translational Research, Caris Life Sciences, Inc., Phoenix, AZ 85040, USA; 3 Division of Radiation Oncology and Cancer Imaging, Department of Oncology, Peter MacCallum Cancer Centre, the University of Melbourne, Melbourne, VIC 3052, Australia; 4 The Lank Center for Genitourinary Oncology, Dana-Farber Cancer Institute, Boston, MA 02215, USA; 5 Department of Hematology and Oncology, University Hospitals Seidman Cancer Center, Cleveland, OH 44106, USA; 6 Department of Oncology, Wayne State University, Karmanos Cancer Institute, Detroit, MI 48201, USA; 7 Department of Hematology and Oncology, University of Minnesota, Masonic Cancer Center, Minneapolis, MN 55455, USA; 8 Clinical Genomics, Hoag Family Cancer Institute, Newport Beach, CA 92663, USA; 9 Department of Translational Molecular Medicine, Saint John’s Cancer Institute, Providence Health Systems, Santa Monica, CA 90404, USA; 10 Division of Medical Oncology, Department of Internal Medicine, College of Medicine, The Ohio State University, The Comprehensive Cancer Center, Columbus, OH 43210, USA; 11 Department of Pathology, Fox Chase Cancer Center, Philadelphia, PA 19111, USA

**Keywords:** renal cell carcinoma, molecular profiling, FOLH1, diagnostics, therapeutics

## Abstract

**Simple Summary:**

While treatments have expanded for advanced renal cell carcinoma (RCC), resistance emerges in the majority of patients. Novel alternative diagnostics and therapeutics are required to improve outcomes. Prostate membrane-specific antigen (PSMA) diagnostic and therapeutic strategies have emerged, and early studies highlight the relevance of PSMA in RCC tumors. These early observations have generated interest in targeting PSMA, which is encoded by the FOLH1 gene, as a diagnostic and therapeutic strategy in RCC. We aimed to investigate patterns of FOLH1 expression in RCC and their impact on outcomes. We identified differential FOLH1 expression based on RCC histology and metastatic sites. We observed a correlation between *FOLH1* expression and an angiogenic gene signature, suggesting potential therapeutic implications for tumors with high *FOLH1* expression. Consistent with our findings, high *FOLH1* expression was associated with an increased time on cabozantinib. Ultimately, this analysis provides insights for designing diagnostic and therapeutic strategies that target *FOLH1*/PSMA.

**Abstract:**

Purpose: Given the emergence of PSMA-targeted diagnostic agents and therapeutics, we sought to investigate patterns of *FOLH1* expression in RCC and their impacts on RCC outcomes. Methods: We conducted a pooled multi-institutional analysis of patients with RCC having undergone DNA and RNA next-generation sequencing. *FOLH1*-high/low expression was defined as the ≥75th/<25th percentile of RNA transcripts per million (TPM). Angiogenic, T-effector, and myeloid expression signatures were calculated using previously defined gene sets. Kaplan–Meier estimates were calculated from the time of tissue collection or therapy start. Results: We included 1,724 patients in the analysis. FOLH1 expression was significantly higher in clear cell (71%) compared to non-clear cell RCC tumors (19.0 versus 3.3 TPM, *p* < 0.001) and varied by specimen site (45% primary kidney/55% metastasis, 13.6 versus 9.9 TPM, *p* < 0.001). *FOLH1* expression was correlated with angiogenic gene expression (Spearman = 0.76, *p* < 0.001) and endothelial cell abundance (Spearman = 0.76, *p* < 0.001). While OS was similar in patients with *FOLH1*-high versus -low ccRCC, patients with *FOLH1*-high clear cell tumors experienced a longer time on cabozantinib treatment (9.7 versus 4.6 months, respectively, HR 0.57, 95% CI 0.35–0.93, *p* < 0.05). Conclusions: We observed differential patterns of *FOLH1* expression based on histology and tumor site in RCC. *FOLH1* was correlated with angiogenic gene expression, increased OS, and a longer duration of cabozantinib treatment.

## 1. Introduction

Renal cell carcinoma (RCC) is a common malignancy among men and women, with increasing incidence over the past decade [1,2]. While most patients present with localized disease, up to 50% of patients with localized RCC develop disease recurrence [3,4]. Even though survival outcomes for patients have improved over the past decade, most patients with advanced or metastatic RCC develop treatment resistance and ultimately lethal disease [5]. Given that the treatment of patients with advanced disease remains suboptimal, alternative diagnostic and treatment approaches are warranted to improve patient outcomes.

Critical to developing innovative technologies in the field is the need to comprehensively molecularly profile RCC, which enables a deeper understanding of diagnostic and therapeutic vulnerabilities. FOLH1, the gene that encodes for prostate-specific membrane antigen (PSMA), a transmembrane glycoprotein, has garnered considerable interest given the advent of new therapeutic agents and diagnostic modalities targeting this protein. While PSMA is expressed in prostate cancer tumors, it is also extensively expressed in various extraprostatic tissues, including but not limited to the salivary glands, liver, gastrointestinal tract, and other endocrine organs, including the breast and adrenal glands [6]. Notably, PSMA is also expressed in tumor-associated neovasculature, and studies on its expression in non-prostate cancer solid tumors have yielded variable results [7].

The expression of PSMA in RCC has essential implications in diagnostic imaging. Currently, computed tomography (CT) and magnetic resonance imaging (MRI) are the main modes of imaging used for initial disease detection, monitoring patients for recurrence post-definitive surgery, and monitoring response to systemic therapy in patients with advanced disease [8]. Historically, metabolic imaging with positron emission tomography (PET) has had a limited role in RCC, mainly due to the physiologic exertion of fluorodeoxyglucose (FDG) from the kidneys and heterogeneity of FDG uptake in renal tumors [9]. There are now novel imaging tracers that target cell surface proteins, including the amino acid L-leucine, PSMA, and, more recently, carbonic anhydrase IX (CAIX) [10]. Emerging evidence may suggest that PSMA PET/CT could be superior to conventional CT imaging for the metastasis detection of RCC tumors [11,12]. These studies highlight the potential for PSMA PET/CT to complement CT in diagnosing and managing RCC, particularly in detecting oligometastatic or oligoprogressive disease that could be amendable to metastasis-directed therapy. While further validation studies are warranted, these studies provide a rationale for investigating *FOLH1* expression across RCC histologic subtypes and sites of metastasis.

In terms of treatment, many PSMA-targeted therapeutics are currently in development and may have potential applications in RCC. These include radioligand therapy, antibody–drug conjugates, bispecific T-cell engagers, chimeric antigen receptor T-cell therapy, and others [13]. In prostate cancer, therapy with the beta-emitting radioligand ^177^Lu-PSMA-617 has demonstrated improved overall survival for patients with metastatic castration-resistant prostate cancer and is now part of the treatment armamentarium for patients with advanced disease [14]. To date, there has been very limited experience with PSMA-directed therapeutics in RCC. However, the implementation of PSMA-targeted therapeutic agents in RCC faces a substantial challenge attributed to the disease’s complex morphologic and genetic heterogeneity [14,15,16]. In light of these dilemmas, there is a need to better characterize the patterns of *FOLH1* expression in RCC and the behavior of tumors with high *FOLH1* expression.

This study aims to characterize the expression patterns of *FOLH1* across variant RCC histology and sites of metastasis and to investigate molecular profiles in tumors with high FOLH1 expression. In addition, we investigate clinical outcomes of patients with tumors with high FOLH1 expression.

## 2. Methods

### 2.1. Study Cohort

The study cohort included patients with a kidney cancer diagnosis (n = 1724) with formalin-fixed paraffin-embedded (FFPE) samples submitted to a commercial CLIA-certified laboratory for molecular profiling (Caris Life Sciences, Phoenix, AZ, USA). Eligible patients included those with a kidney cancer diagnosis with successful sequencing, as described in Section 2.2. No exclusion criteria were applied. All tumor samples categorized with variant histologic characteristics underwent central pathology review by a genitourinary pathologist to confirm the histologic subtype (SW). Tumors classified as mixed subtypes included samples with histologic features of more than one subtype, most commonly papillary with clear cell features, or unspecified features. All tumor samples designated as “RCC, not otherwise specified” were included in all analyses, except those evaluating specific histologic subtypes. The MiT family translocation subtype was confirmed by tumor genomic sequencing. This study was deemed Institutional Review Board exempt, and no patient consent was necessary from the subjects.

### 2.2. DNA/RNA Next-Generation Sequencing

To facilitate the enrichment of tumor elements, microdissection was performed on samples prior to nucleic acid isolation. Next-generation sequencing was performed on isolated genomic DNA using the NextSeq platform (Illumina, Inc., San Diego, CA, USA) for a targeted panel of 592 cancer-relevant genes (N = 497 samples) or the Illumina NovaSeq 6000 platform (Illumina, Inc., San Diego, CA, USA) for whole-exome sequencing (n = 1227 samples). Microdissection was performed prior to sequencing to facilitate the enrichment of tumor elements. Whole-transcriptome sequencing used a hybrid-capture method to pull down the full transcriptome from FFPE tumor samples (n = 1724) using the Agilent SureSelect Human All Exon V7 bait panel (Agilent Technologies, Santa Clara, CA, USA) and the Illumina NovaSeq platform (Illumina, Inc.). Genomic variants were classified by board-certified molecular geneticists according to criteria established by the American College of Medical Genetics and Genomics. When assessing mutation frequencies of individual genes, ’pathogenic’ and ‘likely pathogenic’ variants were counted as mutations, while ‘benign’ and ‘likely benign’ variants and ‘variants of unknown significance’ were excluded.

### 2.3. RNA Expression Analyses

Tumors were characterized as having high or low *FOLH1* expression based on the percentile of RNA transcripts per million (TPM): *FOLH1*-high tumors were in the ≥75th and *FOLH1*-low tumors were in the <25th percentile of expression to allow for more granularity in the data analysis across quartiles of *FOLH1* expression. Angiogenic, T-effector, and myeloid expression signatures were calculated using previously defined gene sets (IMmotion 150 gene signatures) in RCC [17]. Immune cell infiltration in the TME was estimated using MCP-Counter [18].

### 2.4. Immunohistochemistry

Immunohistochemistry (IHC) for PDL1 was performed on FFPE sections of glass slides. Slides were stained using the Agilent DAKO Link 48 (Santa Clara, CA, USA) automated platform and staining techniques, as per the manufacturer’s instructions, and were optimized and validated per CLIA/CAP and ISO requirements. Staining was scored for intensity (0 = no staining; 1+ = weak staining; 2+ = moderate staining; 3+ = strong staining) and staining percentage (0–100%). PD-L1 (SP142)-positive staining was defined as ≥2+ and ≥5% tumor cells.

### 2.5. Tumor Mutational Burden

Tumor mutation burden (TMB) was measured by counting all non-synonymous missense, nonsense, in-frame insertion/deletion, and frameshift mutations found per tumor that had not been previously described as germline alterations in dbSNP151 or the Genome Aggregation Database (gnomAD) or as benign variants identified by Caris’s geneticists. A cutoff point of ≥10 mutations per megabase (mt/MB) was used based on the KEYNOTE-158 pembrolizumab trial [19].

### 2.6. Survival Analysis

Kaplan–Meier survival analysis was used to assess clinical outcomes from insurance claims data. Overall survival was defined as the time from specimen collection to death, censored at the date of last follow up. Time on treatment (TOT) was defined as the time from treatment initiation to treatment discontinuation or death, censored at the date of last follow up.

### 2.7. Statistical Analysis and Data Visualization

Statistical analyses were performed using the python packages Pandas, NumPy, and SciPy. Continuous data were assessed using a Mann–Whitney U test, and categorical data were evaluated using Chi-square or Fisher’s exact test, where appropriate.

## 3. Results

### 3.1. Study Cohort and Patient Characteristics

The cohort included 1724 patients, the majority of whom were male (71%), and the median age at specimen collection was 63 years (Supplement Table S1). *FOLH1* expression was similar among males and females (11.07 versus 11.40 median TPM, respectively, *p* = 0.576) and was not correlated with patient age at the time of profiling (median 63 years, Spearman = −0.01, *p* = 0.700).

### 3.2. FOLH1 Expression across Histologic Subtypes

**Figure 1:**
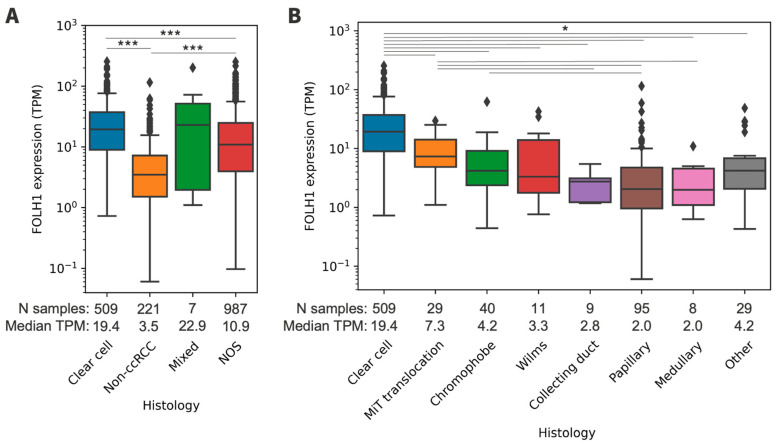
(**A**) *FOLH1* expression across general histologic subgroups. (**B**) *FOLH1* expression across non-ccRCC histologic subtypes. Reference is ccRCC. TPM = transcripts per million; nccRCC = non-ccRCC; NOS = not otherwise specified. * *p* < 0.05, *** *p* < 0.001.

Among histologically classified samples (n = 737), most patients had ccRCC (69%). Samples with non-ccRCC histology (30%) were most commonly papillary RCC (13%), followed by chromophobe RCC (5%) (Supplemental Table S1). *FOLH1* expression was significantly lower in non-ccRCC tumors than in ccRCC tumors (3.48 versus 19.37 TPM, *p* < 0.001) (Supplement Table S2, Figure 1). Additionally, the subset of tumors designated as ‘RCC, not otherwise specified’ (n = 987) had lower *FOLH1* expression compared to that in ccRCC (10.88 versus 19.37 TPM, *p* < 0.001) and likely represented an admixed group of ccRCC and non-ccRCC histopathology.

### 3.3. FOLH1 Expression across Sites of Metastasis

**Figure 2:**
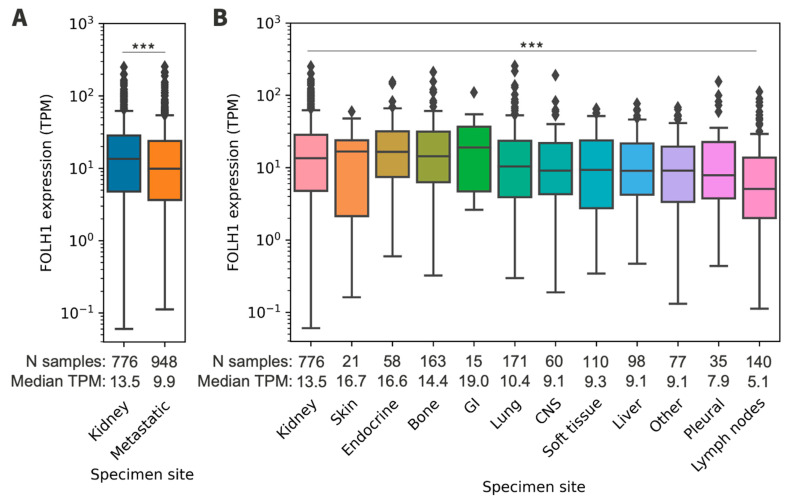
(**A**) *FOLH1* expression in primary kidney and metastatic tumors. (**B**) *FOLH1* expression across sites of metastasis. Reference is the primary kidney. TPM = transcripts per million; GI = gastrointestinal; CNS = central nervous system. *** *p* < 0.001.

The majority of samples were derived from metastatic tissue (55%) and the remainder from the kidney (45%). The most prevalent metastatic sites included the lungs (18%), bones (17%), and lymph nodes (15%). Lymph node tissues were collected from any body site, as samples were not restricted to kidney-draining lymph nodes. *FOLH1* expression varied by specimen site and was lower in metastatic samples compared to the kidney (9.90 versus 13.54 TPM, *p* < 0.001) (Supplement Table S3, Figure 2). Among the metastatic sites, *FOLH1* expression was statistically lower in lymph nodes than in the kidney (5.07 versus 13.54 TPM, *p* < 0.001).

### 3.4. Patterns of DNA Profiling in FOLH1 Expression Groups

**Figure 3:**
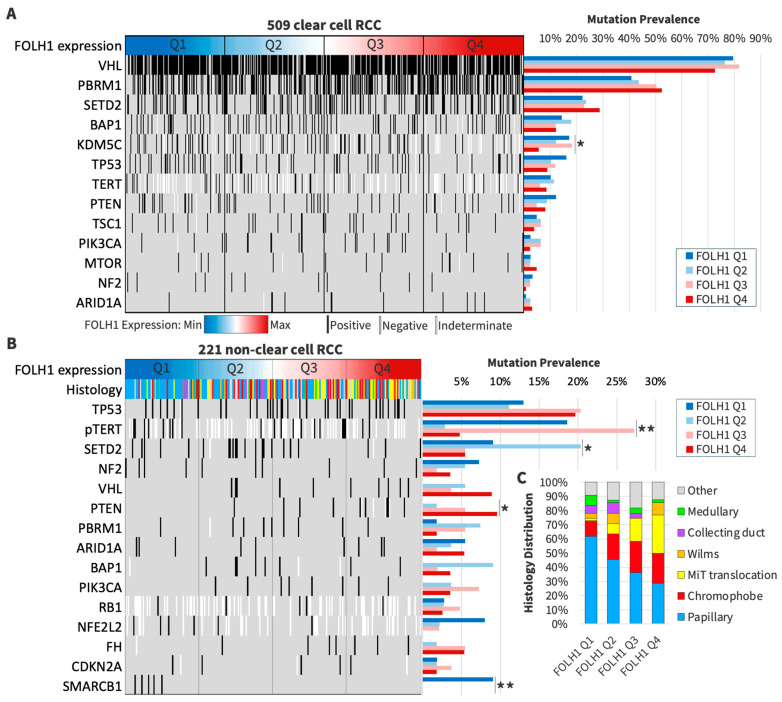
(**A**) Gene mutations associated with *FOLH1* expression quartiles in ccRCC. (**B**) Gene mutations associated with *FOLH1* expression quartiles in non-ccRCC. (**C**) Distribution of histologic types among non-ccRCC *FOLH1* expression quartile subgroups. * *p* < 0.05, ** *p* < 0.01.

To better characterize the histologic groups, we evaluated VHL mutation status among subtype categories: clear cell (77% mutated), non-clear cell (6% mutated), mixed (43% mutated), and not otherwise specified (55% mutated). We characterized the prevalence of gene mutations among subgroups stratified by *FOLH1* expression quartiles for both ccRCC and non-ccRCC. Among both primary and metastatic ccRCC tumors, the most frequently altered genes included *VHL*, *PBRM1*, *SETD2*, *BAP1*, and *KDM5C*, with *KDM5C* mutations less commonly observed among the highest *FOLH1* expression quartile (*FOLH1* Q4: 6% versus 12–18%, *p* < 0.05) (Figure 3A). The most commonly mutated genes among non-ccRCC included *TP53*, *TERT* promoter (*pTERT)*, and *SETD2* (Figure 3B). *PTEN* mutations were absent in tumors with the lowest *FOLH1* expression (Q1) and progressively increased across *FOLH1* expression quartiles (Q2–Q4 range: 2–10%, *p* < 0.05), while *SMARCB1* mutations were exclusively observed in *FOLH1* Q1 (10% vs. 0% Q2–Q3, *p* < 0.01). *pTERT* mutations were more common in *FOLH1* Q1 (19%) than in Q4 (5%, *p* < 0.01) but were most common in Q3 (27%). Higher *FOLH1* expression was less commonly associated with *SETD2* mutations (6% Q4 vs. 9% Q1, *p* < 0.05), with the highest frequency observed in *FOLH1* Q2 (20%). The distribution of histologic subtypes varied across the *FOLH1* expression quartile subgroups, with papillary RCC having decreasing prevalence across increasing *FOLH1* quartiles and MiT translocation RCC having increasing prevalence across increasing *FOLH1* quartiles (Figure 3C).

### 3.5. RNA Signatures and Tumor Microenvironment in FOLH1 Expression Groups

**Figure 4:**
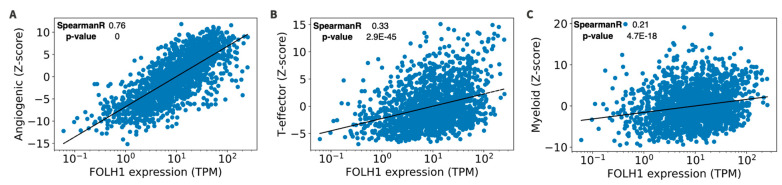
Correlation between *FOLH1* expression and the IMmotion 150 angiogenic (**A**), T-effector (**B**), and myeloid (**C**) gene signatures. TPM = transcripts per million.

**Figure 5:**
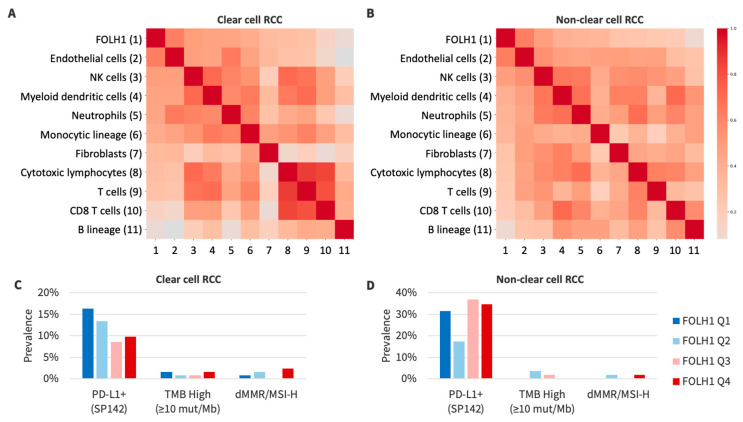
(**A**,**B**) Correlation matrix of *FOLH1* expression and relative abundance of immune/stromal cell populations in the tumor microenvironment in ccRCC (**A**) and non-ccRCC (**B**), with the matrix sorted by correlations with *FOLH1* expression. (**C**,**D**) Prevalence of biomarkers of response to immunotherapy, including PD-L1+ (SP142 IHC; ≥2+ staining in ≥5% of cells), tumor mutation burden-high (TMB-High; ≥10 mutations/Mb), and deficient mismatch repair/microsatellite instability-high (dMMR/MSI-H) among ccRCC (**C**) and non-ccRCC (**D**) tumors.

*FOLH1* expression was strongly correlated with the IMmotion 150 angiogenic gene signature (Spearman = 0.76). There was a weak correlation between *FOLH1* expression and the T-effector gene signature (Spearman 0.33) and myeloid gene signature (Spearman 0.21) (Figure 4). We correlated components of the tumor microenvironment with *FOLH1* expression. There was a strong correlation between endothelial cell abundance (Spearman 0.76) in the tumor microenvironment and *FOLH1* expression, with weaker correlations for immune cell types (Spearman 0.04–0.50) (Figure 5). PD-L1+ frequency was numerically lower yet not significantly different in *FOLH1*-high compared to *FOLH*-low tumors among patients with ccRCC (10% versus 17%, respectively, *p* = 0.07) but was similar among non-ccRCC tumors (31% versus 32%, respectively, *p* = 0.95).

### 3.6. Overall Survival by FOLH1 Expression

**Figure 6:**
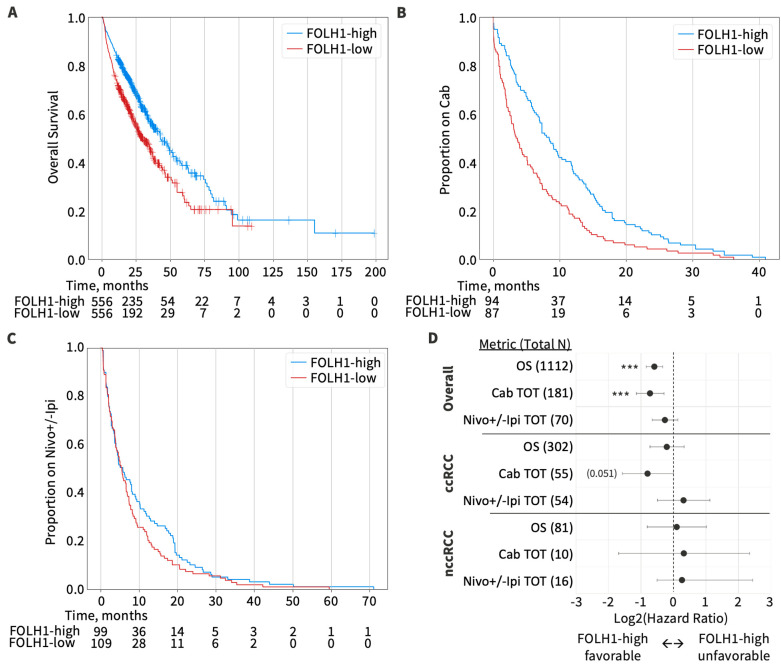
(**A**) Kaplan–Meier plot of OS for the overall cohort stratified by the median *FOLH1* expression. (**B**) Kaplan–Meier plots of cabozatinib (Cab) TOT for the overall cohort stratified by the median *FOLH1* expression. (**C**) Kaplan–Meier plots of nivolumab with or without ipilimumab (Nivo+/-Ipi) TOT for the overall cohort stratified by the median *FOLH1* expression. (**D**) Forest plot of OS, Cab TOT, and Nivo+/-Ipi TOT for the overall cohort, ccRCC subgroup, and non-ccRCC subgroup. *** *p* < 0.001.

We evaluated OS in cohorts stratified by the median *FOLH1* expression (Figure 6). Among all RCC tumors with available clinical data (n = 1112), the median OS in *FOLH1*-high vs. -low RCC tumors was 42.8 versus 30.0 months (HR 0.67, 95% CI 0.57–0.80, *p* < 0.001). Patients with ccRCC *FOLH1*-high tumors had a median OS of 48.4, versus 42.6 months in *FOLH1*-low patients (HR 0.87, 95% CI 0.61–1.26, *p* = 0.469). In patients with non-ccRCC, the median OS in *FOLH1*-high versus -low RCC tumors was not significant; 30.2 vs. 30.0 months (HR 1.08, 95% CI 0.57–2.03, *p* = 0.817).

Additionally, we evaluated whether *FOLH1* expression was associated with differences in cabozantinib (no concurrent immunotherapy) and nivolumab with or without ipilimumab TOT given in any line of therapy. Cabozantinib was selected because it was the most frequently utilized tyrosine kinase inhibitor (TKI) in the database. Nivolumab +/− ipilimumab was selected because it was the most frequently utilized pure immune checkpoint inhibitor regimen in the database. In the overall cohort, patients with *FOLH1*-high tumors had a longer cabozantinib TOT than patients with *FOLH1*-low tumors (7.4 versus 3.7 months, respectively, HR 0.61, 95% CI 0.45–0.82, *p* < 0.0001), while nivolumab +/− ipilimumab TOT was similar between *FOLH1* groups (5.8 versus 5.5 months, respectively, HR 0.84, 95% CI 0.64–1.10, *p* = 0.205). When evaluating outcomes by histologic subsets, patients with *FOLH1*-high ccRCC had a longer cabozantinib TOT (9.7 versus 5.2 months, respectively, HR 0.58, 95% CI 0.34–1.01, *p* = 0.051), while there was no significant difference in nivolumab +/− ipilimumab TOT between *FOLH1* groups (4.6 versus 4.0 months, respectively, HR 1.25, 95% CI 0.71–2.18, *p* = 0.428). Among non-ccRCC, patients with *FOLH1*-high and -low tumors had similar cabozantinib TOT (8.1 versus 1.4 months, HR 1.26, 95% CI 0.31–5.1, *p* = 0.748) and nivolumab +/− ipilimumab TOT (3.0 versus 4.3 months, HR 1.21, 95% CI 0.43–3.38, *p* = 0.734).

## 4. Discussion

In this study, we comprehensively characterized *FOLH1* expression across RCC tumors and evaluated the impact of *FOLH1* expression on disease outcomes. To our knowledge, this is the largest study to date investigating *FOLH1* expression in RCC, with a dataset comprising 1,724 patients having undergone in-depth molecular profiling. This analysis identifies several novel insights that inform our understanding of *FOLH1* expression in RCC. We demonstrate that *FOLH1* expression is variable across RCC histopathologic types, with increased expression in ccRCC compared to non-ccRCC. Additionally, we demonstrate differential *FOLH1* expression patterns across sites of metastasis, with decreased expression in metastases compared to primary tumors. This finding may imply potentially worse treatment response to anti-VEGF therapy in metastatic disease. Additionally, this finding implies that lower *FOLH1* expression in metastatic tissue may yield variable results with PSMA PET imaging in the metastatic setting. Comprehensive RNA analysis highlights the correlation of *FOLH1* expression with an angiogenic gene signature and increased endothelial cell abundance in the tumor microenvironment, suggesting potential therapeutic implications for tumors with high *FOLH1* expression. In line with this hypothesis, we show that tumors with high *FOLH1* expression had extended time on cabozantinib treatment. With the advent of PSMA-targeted diagnostic and therapeutic treatments, this comprehensive data provide a scaffold for the rationale to investigate PSMA-targeted diagnostic and therapeutic strategies in RCC.

In our study, we demonstrated higher *FOLH1* expression in clear cell compared to variant-histology tumors. Prior studies have evaluated PSMA IHC in RCC and, to a more limited extent, *FOLH1* RNA expression, using next-generation sequencing. Lopes et al. were the first to describe PSMA expression in proximal renal tubular cells [20]. Since this report, several subsequent studies have evaluated PSMA expression in primary RCC tumors and RCC cells in circulation [21,22,23,24,25]. The majority of these studies employed IHC to assess PSMA protein expression, and most reported PSMA within the neovasculature associated with the tumor as opposed to tumor cells directly. Baccala and colleagues were among the first to describe PMSA expression across variant histologic types of RCC (n = 109). In this study, PSMA expression in tumor-associated neovasculature was greatest among ccRCC tumors (76%), followed by renal oncocytoma (53%) and chromophobe RCC (31%). No tumor with papillary RCC demonstrated positive PSMA staining [26]. Similarly, Al-Ahmadie et al. demonstrated strong PSMA expression in 80% of ccRCC tumors, with no staining in papillary RCC [7]. More recently, Spatz and colleagues confirmed these results in a series where vascular expression of the FOLH1 gene was assessed in 257 patients with RCC from The Cancer Genome Atlas [27]. These studies have been supported by imaging studies demonstrating increased PSMA uptake on PET in ccRCC [9,10,11,16,28]. These findings are significant, considering the potential clinical application of PET imaging in RCC detection. While a comprehensive review of 93 studies revealed the diagnostic value of radiomics in the differentiation of RCC [29], another systematic review including 331 patients concluded that PSMA-targeted PET/CT imaging was associated with enhanced detection in ccRCC and could be utilized as a biomarker of disease aggressiveness [30]. Additionally, a retrospective study of 61 patients who underwent PSMA PET/CT for restaging or suspected metastatic RCC demonstrated PSMA PET-positive disease in 84% of patients, resulting in a change in management in 49% of patients [31].

Studies of PSMA PET imaging in non-ccRCC have been limited. A small series of eight patients with non-ccRCC lacking histopathologic correlation demonstrated that only 14% of lesions had high radiotracer avidity [32]. Although the differential patterns of *FOLH1* and PSMA expression across tumor subtypes of ovarian, cervical, breast, and colorectal cancer have been evaluated, there remains a gap in the literature regarding the comparative analysis of *FOLH1* RNA among subtypes of RCC. In consideration of previous studies, highlighting the diagnostic potential of liquid biopsy biomarkers in RCC [33], the combination of tissue-based molecular profiling and liquid biopsy biomarkers could be considered a promising future direction for achieving more precise diagnostics in RCC. Our study was restricted to the molecular analysis of tissue alone, which is a limitation, as additional novel blood-, imaging-, and pathology-based biomarkers, some of which integrate reliance on artificial intelligence methodologies, are currently being developed. For example, a blood-based biomarker that integrates baseline and dynamic changes in *FOLH1* at the time of clinical treatment decision would have increased utility in guiding clinical decision making. Larger confirmatory studies integrating molecular characterization are warranted to help develop a niche for PSMA-targeted diagnostics and therapeutics in RCC. Our study confirms these results, with the highest *FOLH1* expression seen in ccRCC and lower expression in papillary RCC, which had the second lowest median TPM of all the histologic tumors evaluated. These observations have implications for the application of PSMA-targeted diagnostic and therapeutic strategies, with rationale for increased applications in ccRCC.

A novel aspect of our study is the evaluation of *FOLH1* expression across sites of metastasis for patients with RCC. To date, a comprehensive assessment of *FOLH1* expression across sites of metastasis is lacking, and PSMA IHC expression studies have largely been limited to the assessment of nephrectomy tumor specimens. Additionally, more recent PSMA PET imaging studies have not systematically characterized PSMA uptake patterns across sites of metastasis in RCC, and no study has integrated a histopathologic correlation. While RCC is regarded as a vascular tumor, we hypothesized that less vascular metastatic sites would express lower *FOLH1* and, conversely, more vascular sites would express higher levels. In our study, we in fact observed differential *FOLH1* expression patterns across sites of metastasis. While lymph node metastases had significantly decreased expression compared to the kidney, *FOLH1* expression in the bone, endocrine glands, skin, and gastrointestinal tract was numerically higher than that in the kidney. Given that the site of metastasis is a prognostic factor in RCC, with bone associated with worse outcomes and endocrine metastases associated with improved prognosis [34,35], these findings have implications for therapeutic strategies in the advanced setting based on the predominance of metastatic sites involved.

In our study, we evaluated mutated genes across quartiles of *FOLH1* expression in both clear cell and non-clear cell RCC. In general, for patients with ccRCC, we demonstrated similar mutation prevalence across quartiles of *FOLH1* expression, with the exception of mutations in *KDM5C,* which were less commonly observed in tumors with higher *FOLH1* expression. *KDM5C* is a histone demethylase gene linked with glycogen metabolism and the regulation of several hypoxia-inducible factor (HIF)-related genes [36]. Mutations in *KDM5C* have recently been associated with increased tumor angiogenesis and longer progression-free survival on sunitinib [37]. Concurrent expression of *KDM5C* and *PBRM1*, which encodes a subunit of the SWI/SNF chromatin remodeling complex, is present in up to 10% of RCC tumors, enriched in patients with favorable risk disease, and associated with longer time on VEGF TKI therapy [38].

Our RNA analysis identified a strong correlation between *FOLH1* expression and the IMmotion 150 angiogenic gene signature. This signature has been associated with improved outcomes on VEGF TKI treatment, suggesting that *FOLH1* expression or potentially PSMA expression on IHC or uptake on PET imaging could be investigated as a biomarker of response to cabozantinib. While in our study, microdissection was performed prior to sequencing to facilitate the enrichment of tumor elements, we were not able to distinguish between the tumor cells and tumor-associated neovasculature *FOLH1* expression. The observation of endothelial cell predominance in the tumor microenvironment aligns with prior observations of PSMA IHC expression in the tumor-associated neovasculature [21,22,23,24,25]. Taken together, we suggest a possible explanation for the relationship between FOLH1 expression and improved outcomes of cabozantinib treatment, indicating a pathway involving PSMA localization on endothelial cells. Our analysis did not demonstrate any significant association of *FOLH1* with immune signatures, tumor-infiltrating immune cells, or PD-L1. To our knowledge, this association has not been demonstrated in other tumor types. However, *FOLH1* and PSMA expression have been studied in ovarian, cervical, breast, and colorectal cancers [39,40,41]. Future studies integrating single-cell RNA sequencing and spatial profiling technologies will be critical to better profiling the components of the tumor microenvironment. Our findings are hypothesis-generating and, if validated in larger studies that more directly integrate clinical data, could help guide the design of future biomarker studies that utilize FOLH1 as an integrated biomarker to guide angiogenic/VEGF-based treatment strategies for patients with RCC and potentially other solid tumor malignancies.

Lastly, we investigated the impact of *FOLH1* expression on survival outcomes. We hypothesized that *FOLH1*-high tumors would be associated with improved overall survival, as these tumors are more angiogenic and typically associated with improved prognosis [17]. We found that high *FOLH1* expression was associated with longer overall survival for the entire cohort. However, there was no difference in survival base on *FOLH1* status within the ccRCC and non-ccRCC subpopulations. This observation is likely the result of the enrichment of FOLH1 in ccRCC; thus, analysis by FOLH1-high and -low tumors in the overall cohort largely reflected a comparative analysis between ccRCC and nccRCC, which historically portends a worse progression [42]. In an analysis by Spatz and colleagues from The Cancer Genome Atlas, *FOLH1* mRNA expression was associated with worse OS in pT2 and higher-stage tumors in univariate analysis but not multivariable analysis [27]. In our study, data regarding the stage at diagnosis were lacking, and our cohort consisted of predominately tumors from metastatic sites. Given the overwhelming evidence of enhanced angiogenesis in *FOLH1*-high tumors, we investigated the activity of VEGF-targeted therapies in *FOLH1*-high and -low tumors. We observed improved TOT with cabozantinib in *FOLH1*-high tumors. For comprehensive assessment, we evaluated TOT with nivolumab with or without ipilimumab and did not observe differences in outcomes among FOLH1-high and -low groups. These hypothesis-generating data suggested that *FOLH1* expression could be used as a biomarker to predict sensitivity to therapeutic strategies targeting the VEGF pathway and angiogenesis. Studies on PSMA-targeting therapies in RCC have been limited. Milowsky and colleagues reported the results of a phase 1 study of J591, a monoclonal antibody targeting PSMA, in advanced solid tumors, which included 10 patients with RCC [43]. While no objective responses were observed, the proof-of-principal study confirmed the ability to direct a ligand to a neovasculature-specific target. In a case report by Zhang and colleagues of a single patient with RCC treated with ^177^Lu-PSMA-I&T, post-treatment whole-body scintigraphy and SPECT/CT demonstrated rapid washout from the tumor [44]. These data suggest that current radiotracers may not be suitable theranostic agents in RCC, and different agents with longer tumor retention may have the potential for enhanced activity in RCC. Additional prospective studies with integrated tissue, blood, imaging, and clinical correlative analyses will be critical in defining the role of PSMA-based therapeutic strategies such as radioligand therapy in RCC and their impact on future directions for combination therapy strategies involving bispecific antibodies and antibody–drug conjugates. Our data provide a rationale for the investigation of combined PSMA- and VEGF/HIF-targeting agents and highlight the future clinical potential of molecular biomarker-driven therapy decisions in RCC. Prospective studies must include potential diagnostic and therapeutic biomarkers in guidelines.

Although this is the most extensive study to date dissecting the impact of *FOLH1* expression on RCC, several limitations exist. While our cohort was similar in age and gender to the real-world RCC population, we lacked granular clinical data regarding tumor stage, grade, and prognostic risk groups. Furthermore, a comparative analysis of *FOLH1* expression based on pathologic characteristics such as nuclear grade or the presence of sarcomatoid/rhabdoid features was precluded due to the unavailability of corresponding data. We were also unable to control for potential confounding clinical variables in our survival analysis due to the retrospective nature of this study. Another significant limitation was the lack of histological annotation for the cohort of patients classified as ‘RCC, not otherwise specified’ on local institutional pathologic assessment. The assessment of *VHL* status in these subgroups suggests that this is likely an admixed population with predominately ccRCC. Furthermore, we utilized TOT as a surrogate for progression-free survival, as granular data regarding radiographic response to treatment and reason for treatment discontinuation were lacking. Additionally, another limitation of this work as the restriction to tissue-based DNA, RNA, and limited protein-based markers. The integration of blood-, additional tissue-, imaging-, and pathologic-based biomarkers will provide a more comprehensive evaluation of *FOLH1* and the potential dynamic changes over time under the selective pressures of treatment. Such future studies are recommended to more broadly understand *FOLH1* in RCC and potential implications for treatment.

## 5. Conclusions

In summary, in this study, we comprehensively dissected the patterns of *FOLH1* expression across RCC tumors and evaluated the impact of *FOLH1* expression on disease outcomes. As the largest cohort study of molecularly profiled RCC tumors investigating *FOLH1* expression, this analysis provides important insights for designing diagnostic and therapeutic treatment strategies in RCC that target *FOLH1* and PSMA. Additional studies with expanded clinical annotation and spatial profiling will be critical for improving our ability to rationally design enhanced treatment strategies that target *FOLH1* as a potential therapeutic vulnerability in RCC. Ultimately, prospective clinical trials will be warranted to better determine the utility of our molecular findings.

## Data Availability

Data are available upon reasonable request.

## Supplementary Materials

The following supporting information can be downloaded at: https://www.mdpi.com/article/10.3390/cancers16101855/s1, Table S1: Patient demographics; Table S2: FOLH1 expression across histologic subtypes. Reference is clear cell RCC; Table S3: FOLH1 Expression Across Sites of Metastasis.
