# Supplementary material for: Characterization of FOLH1 Expression in Renal Cell Carcinoma
Source: Cancers (Basel). 2024 May 13;16(10):1855. doi: 10.3390/cancers16101855 (PMC11119455; doi:10.3390/cancers16101855)
Supplement: Supplementary file 1 [file cancers-16-01855-s001.zip › cancers-2974520-supplementary.pdf]

Supplement Table S1. Patient demographics.

Cohort Characteristics			
**Age**	**Median (range)**	**Correlation *FOLH1* expression (TPM)**	**P-value**
Years at tissue collection	63 (1-90+)	-0.01 (Spearman)	0.70
**Sex**	**N (%)**	**Median *FOLH1* expression (TPM)**	**P-value**
Male	1221 (71%)	11.4	0.58
Female	503 (29%)	11.1	

TPM=Transcripts per million.

Supplement Table S2. *FOLH1* expression across histologic subtypes. Reference is clear cell RCC.

Histology	N (%)	Median *FOLH1* expression (TPM)	P-Value*
**ccRCC**	509 (69%)	19.4	-
**Non-ccRCC**	221 (30%)	3.5	<0.001
Papillary	95 (13%)	2.0	<0.001
Chromophobe	40 (5%)	4.2	<0.001
MiT Translocation**	29 (4%)	7.3	<0.001
Wilm's tumor	11 (2%)	3.3	0.01
Collecting duct	9 (1%)	2.8	<0.001
Medullary	8 (1%)	2.0	<0.001
Other	29 (4%)	4.2	<0.001
**Mixed**	7 (1%)	22.9	0.77
**Not otherwise specified**	987 (----%)	10.9	<0.001

TPM=Transcripts per million.
*In reference to clear cell RCC.
**Molecularly defined.

Supplement Table S3. *FOLH1* Expression Across Sites of Metastasis

Biopsy site	N (%)	Median *FOLH1* expression (TPM)	P-value*
**Kidney**	776 (45%)	13.5	-
**Metastatic**	948 (55%)	9.9	<0.001
**Lung**	171 (18%)	10.4	0.18
**Bone**	163 (17%)	14.4	0.37
**Lymph nodes**	140 (15%)	5.1	<0.001
**Soft tissue**	110 (12%)	9.3	0.07
**Liver**	98 (10%)	9.1	0.10
**Other**	77 (8%)	9.1	0.07
**CNS**	60 (6%)	9.1	0.27
**Endocrine**	58 (6%)	16.6	0.37
**Pleural**	35 (4%)	7.9	0.32
**Skin**	21 (2%)	16.7	0.43
**Gastrointestinal**	15 (2%)	19.0	0.67

TPM=Transcripts per million; CNS=Central nervous system.
*Reference is the kidney.
